# Refusal to take a sick leave as an estimate of the phenomenon of presenteeism in Poland

**DOI:** 10.18632/oncotarget.25592

**Published:** 2018-06-15

**Authors:** Grzegorz Juszczyk, Aleksandra Czerw, Anna Augustynowicz, Tomasz Banaś, Marcin Mikos, Urszula Religioni, Andrzej Deptała

**Affiliations:** ^1^ Department of Public Health, Medical University of Warsaw, Warsaw, Poland; ^2^ Department of Health Economics and Medical Law, Medical University of Warsaw, Warsaw, Poland; ^3^ Department of Economic and System Analyses, National Institute of Public Health–NIH, Warsaw, Poland; ^4^ Department of Gynecology and Oncology, Jagiellonian University Medical College, Cracow, Poland; ^5^ Department of Medicine and Health Sciences, Modrzewski Cracow Academy, Cracow, Poland; ^6^ Department of Cancer Prevention, Medical University of Warsaw, Warsaw, Poland

**Keywords:** absenteeism, presenteeism, prevalence of presenteeism, health problems

## Abstract

**Introduction:**

Absenteeism and presenteeism are two main phenomena related to health problems and professional activity. Presenteeism is the involvement in a professional activity despite being ill. The purpose of the current study is to estimate the prevalence of presenteeism in Poland on the basis of medical records and to explore associations between presenteeism and patients’ age, gender and type of medical problem. Another purpose is to provide estimates of the length of sick leave if it was accepted.

**Results:**

The amount of patients who refused to take a sick leave was 27.4%. There was a minor relationship between the refusals and gender (slightly higher in men) as well as strong effects of the age of patients (periods of sick leave were longer in older patients) and ICD-10 diagnosis (largely in acute diseases of the upper respiratory tract). The estimated number of days spent on sick leave in the group of patients that refused to take it, assuming that they made a different decision and complied to it, was in the range between 5 and 10 days.

**Discussion:**

The prevalence of presenteeism in Poland is relatively high. Since the largest proportion of refusals took place in the case of potentially contagious diseases, the negative impact on productivity may be even higher. Even though the relationship between presenteeism and wages remains unclear, the remarkable increase of wages in Poland within the last 20 years may explain the propensity to work despite being ill. Further research needs to consider the simultaneous use of medical records and self-measured productivity loss.

**Materials and Methods:**

The current study is based on data from medical records concerning 550,360 patients aged 19–64. Associations between refusals to take a sick leave and patients’ age, gender, as well as diagnosis in terms of ICD-10 (International Statistical Classification of Diseases and Related Health Problems), were tested. Linear regression analysis on the data acquired from the patients who accepted to take a sick leave were further used to estimate the possible length of sick leave in the group of patients that refused to take it.

## INTRODUCTION

The problem of absenteeism, its costs and correlations has been well described in scientific literature [1–4]. Apart from sickness, another phenomenon related to being sick and continuing professional activity is sickness presence. Furthermore, presenteeism means to be professionally active, i.e. attending work or running a business, despite being ill. Some reports estimate that the costs of presenteeism are actually higher than the costs of absenteeism [5, 6]. Together with the direct costs of health care, absenteeism and presenteeism, which lead to productivity loss, are the three main categories of employers’ costs related to the employee health status. Estimates on the proportion of the costs of presenteeism range from 18% to 61% [7]. The costs of presenteeism in terms of reduced professional efficiency in the USA were estimated to reach 150 billion dollars per year [8].

It was also proved that presenteeism might be a problem in circumstances where absenteeism is not [9]. The clinical group, e.g. people suffering from migraine, may not differ from the control group in terms of presenteeism and, at the same time, it may differ in terms of productivity loss while being present at work. Several days of presenteeism may also worsen health and cause absenteeism [10].

Depending on the reason for being ill, which can be acute, episodic or chronic, and the type of job, the consequences of presenteeism include a decrease in the quantity and/or the quality of work. Difficulties associated with concentration, persistent distraction, fatigue and irritability are the main hindering factors. The difficulties are attributable to both health conditions as well as medication or treatment that is carried out. If infectious diseases are at stake, there is also the risk of spreading the illness.

Some jobs (e.g. care giving for the elderly) may demand attendance more than others. Also, if the pace of work is not so important or it is not controlled, presenteeism is more prevalent [11]. The level of presenteeism is also higher when there is no possibility for replacement and the necessary work accumulates until the ill person returns [12]. A sense of loyalty for colleagues when working in a team also strengthens presenteeism [13]. Presenteeism is negatively correlated with job satisfaction and positively correlated with stress at work and professional burnout [14, 15].

A theory of presenteeism summarizing the mutual relationships between adequate variables discussed above is being developed [16]. It contains theorems about relationships and possible interactions, e.g. how presenteeism translates into productivity loss and how personality traits, attitudes and organizational factors possibly may moderate this effect.

The purpose of the current study is to estimate the prevalence of presenteeism in Poland on the basis of medical records as well as to explore possible associations between presenteeism and patients’ age, gender and type of medical problem. The prevalence of presenteeism will be interpreted in the context of current diagnosis and the estimated length of sick leave in the case of patients who decided to take advantage of the doctor's recommendation and cease professional activity until the end of their recovery.

## RESULTS

### The sample characteristics

Table 1 presents the frequency distribution of the patients’ age.

**Table 1:** Frequency distribution of the patients’ age

Age	*n*	*%*
19–24 years	42,214	7.7
25–29 years	125,784	22.9
30–34 years	135,554	24.6
35–39 years	92,192	16.8
40–44 years	55,563	10.1
45–49 years	31,630	5.7
50–54 years	24,754	4.5
55–59 years	27,678	5.0
60–64 years	14,991	2.7
Total	550,360	100

*n* - number of participants; *%* - percentage of the sample.

In most cases, the patients’ age was between 30 and 34.

Table 2 presents the frequency distribution for diagnoses in terms of ICD-10 chapters.

**Table 2:** Frequency distribution of diagnoses in terms of ICD-10 chapters

Chapter	*n*	*%*
Certain infectious and parasitic diseases (A00-A99)	12,109	2.200
Certain infectious and parasitic diseases (B00-B99)	4,137	0.752
Neoplasms (C00-D48)	700	0.127
Diseases of the blood and blood-forming organs and certain disorders involving the immune mechanism (D50-D89)	2,150	0.391
Endocrine, nutritional and metabolic diseases (E00-E90)	4,640	0.843
Mental and behavioral disorders (F00-F99)	9,757	1.773
Diseases of the nervous system (G00-G99)	10,959	1.991
Diseases of the eye and adnexa (H00-H59) and Diseases of the ear and mastoid process (H60-H95)	9,602	1.745
Diseases of the circulatory system (I00-I99)	8,036	1.460
Diseases of the respiratory system (J00-J99)	284,529	51.699
Diseases of the digestive system (K00-K93)	21,739	3.950
Diseases of the skin and subcutaneous tissue (L00-L99)	4,332	0.787
Diseases of the musculoskeletal system and connective tissue (M00-M99)	46,969	8.534
Diseases of the genitourinary system (N00-N99)	11,811	2.146
Pregnancy, childbirth and the puerperium (O00-O99)	39,432	7.165
Certain conditions originating in the perinatal period (P00-P96)	5	0.001
Congenital malformations, deformations and chromosomal abnormalities (Q00-Q99)	117	0.021
Symptoms, signs and abnormal clinical and laboratory findings, not classified elsewhere (R00-R99)	30,987	5.630
Injury, poisoning and certain other consequences of external causes (S00-S99)	24,688	4,895
Injury, poisoning and certain other consequences of external causes (T00-T98)	4.486	0.889
External causes of morbidity and mortality (V01-V99)	129	0.023
External causes of morbidity and mortality (W00-W99)	519	0.094
External causes of morbidity and mortality (X00-X99)	50	0.009
External causes of morbidity and mortality (Y00-Y98)	67	0.012
Factors influencing health status and contact with health services (Z00-Z99)	18,001	3.271

*n* - number of participants; *%* - percentage of the sample.

The majority of cases were diagnosed with a group-J disease, i.e. diseases of the respiratory system.

### Refusals in response to doctor's recommendations on sick leave

In the group of 550,360 patients, there were 150,720 patients (27.4%) who refused to take a sick leave. The remaining part of the sample, i.e. 399,640 patients (72.6%), were on sick leave for a period lasting from 1 to 182 days (*M* = 7.75; *SD* = 8.34), which is the maximal length of paid sick leave in Poland.

Table 3 presents the frequency distribution of the sick leave refusals in the group of women and in the group of men.

**Table 3:** Frequency distribution of sick leave refusals in the group of women and in the group of men

	Women		Men		Total	
Sick leave	*n*	%	*n*	%	*n*	%
Complying	250,913	73.6	148,727	71.0	399,640	72.6
Refusal	89,846	26.4	60,874	29.0	150,720	27.4
Total	340,759	100	209,601	100	550,360	100

*n* - number of participants; *%* - percentage of the sample.

On the basis of the chi-square independent test, the relationship between gender and sick leave refusal was found to be statistically significant, χ^2^(1) = 467.45, *p* < .001. The number of patients who decided to refuse to take a sick leave was slightly higher in the group of men than in the group of women. However, the effect size in terms of Cramer's V coefficient was weak, *V* = .03, *p* < .001.

**Figure 1:**
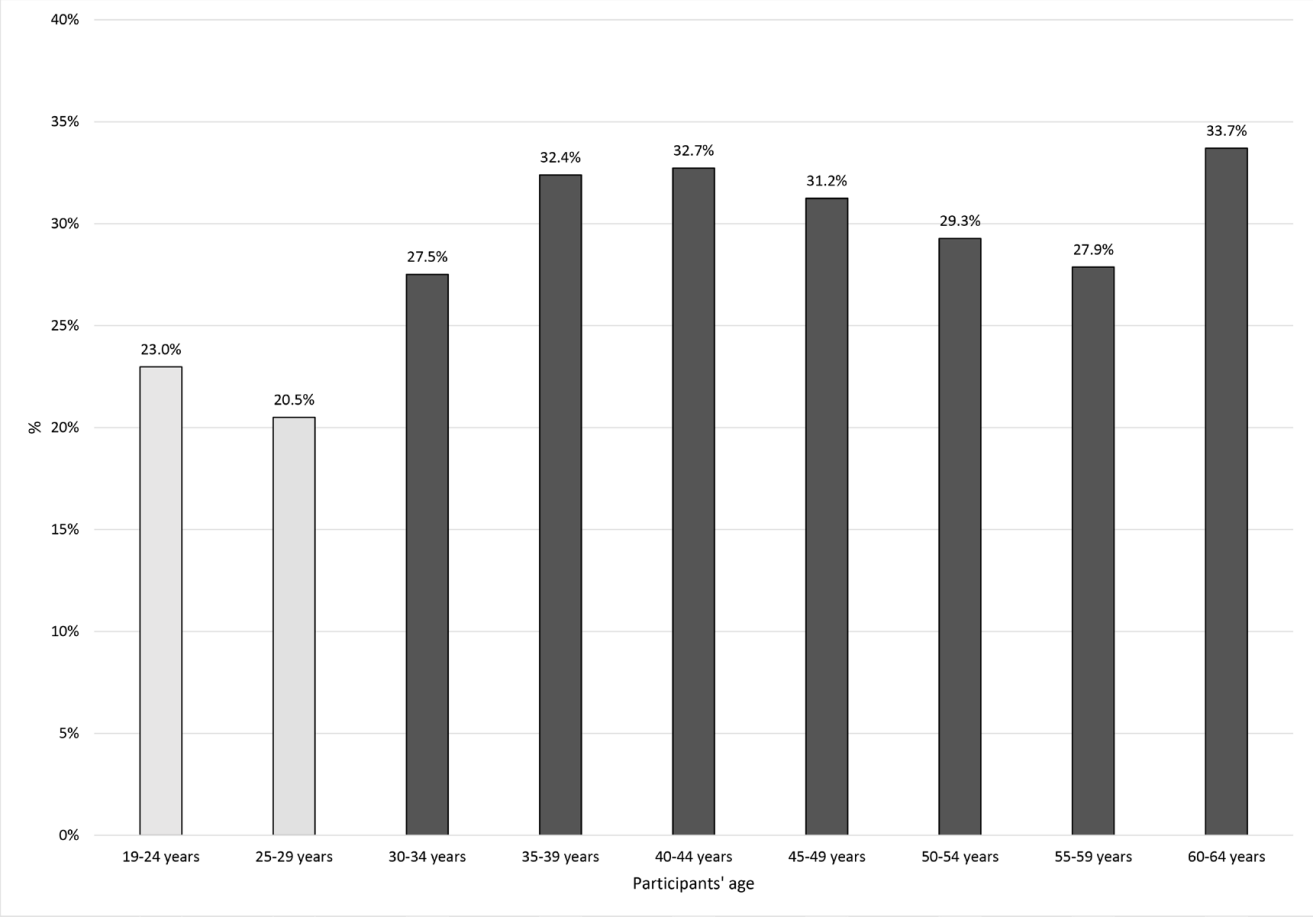
The association between refusal to take a sick leave and patients’ age

Also, on the basis of the chi-square independent test, the relationship between the participants’ age and sick leave refusal was found to be statistically significant, χ^2^(8) = 5952.74, *p* < .001. The number of patients who decided to refuse to take a sick leave was lower in the group of patients aged 19–24 and in the group of patients aged 25–29 (see Figure 1). The effect size in terms of Cramer's V coefficient was moderate, *V* = .10, *p* < .001.

**Figure 2:**
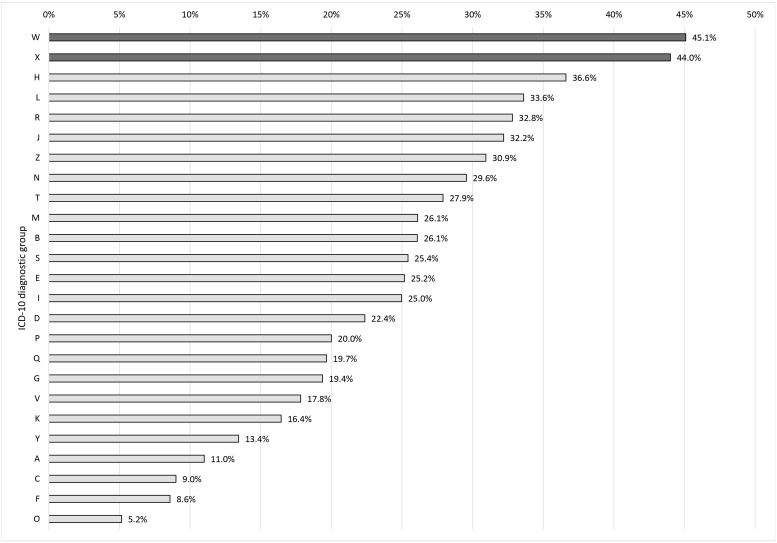
The association between the refusal to take a sick leave and diagnosis in terms of ICD-10 diagnostic group

There was also a statistically significant relationship between diagnoses in terms of ICD-10 chapters and refusal to take a sick leave, χ^2^(24) = 19620.79, *p* < .001. The effect size in terms of Cramer's V measure was strong, *V* = .19, *p <* .001. Figure 2 presents the frequency distribution for refusals in the groups of diseases. The categories were sorted from the one associated with the most number of refusals to the one associated with the least number of refusals. The refusals were most prevalent in cases with diagnoses from groups W and X, i.e. accidents caused by external factors (represented by the dark bars).

**Figure 3:**
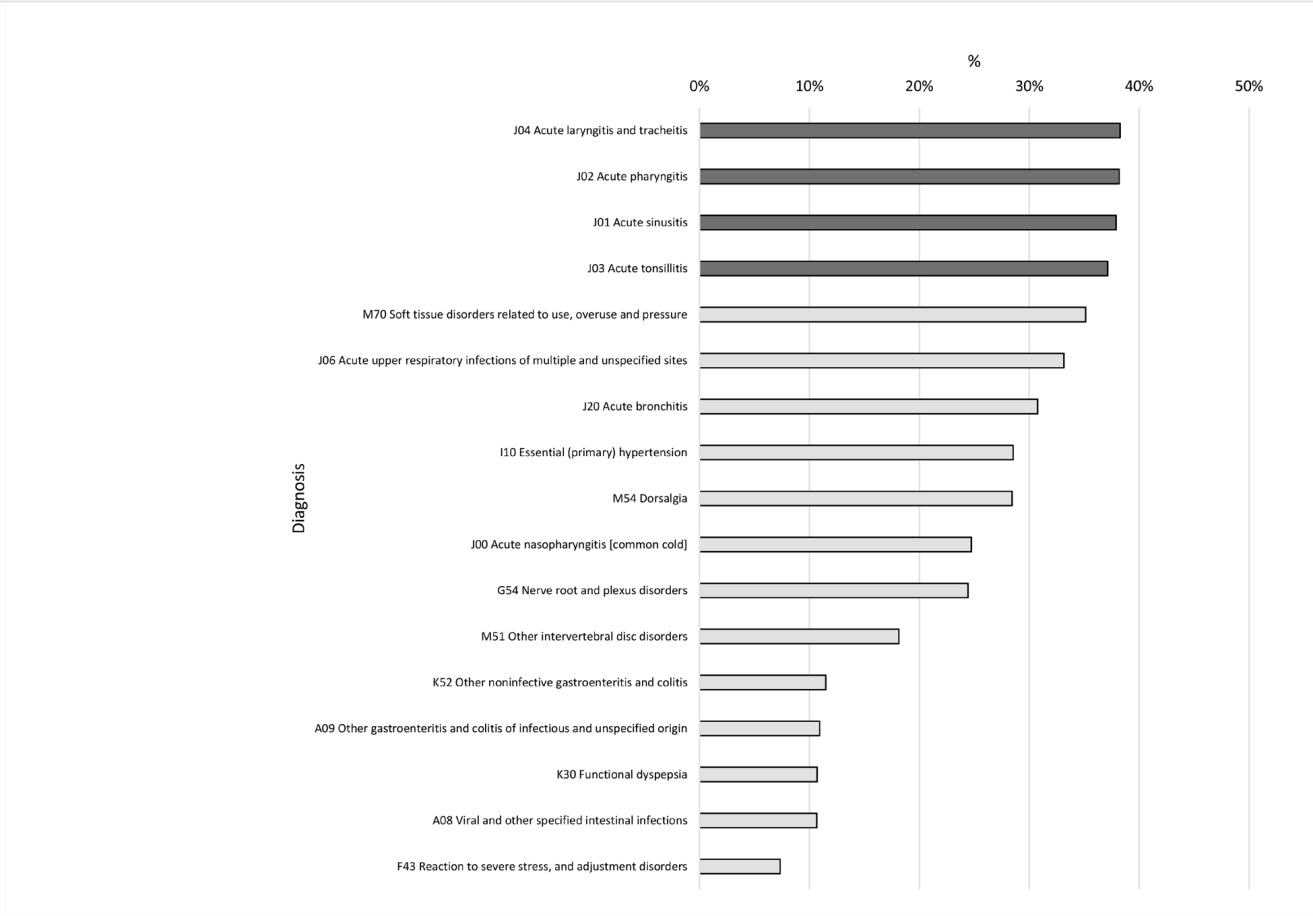
The association between the refusal to take a sick leave and diagnosis in terms of ICD-10 diagnostic codes

The association between diagnosis in terms of ICD-10 codes and sick leave refusal was also statistically significant, χ^2^(16) = 8525.96, *p* < .001. The effect was strong, *V* = .16, *p* < .001. The analysis included diagnoses that were made in at least 4.000 cases. Figure 3 presents the frequency distributions for diagnostic categories sorted from the ones that were associated with the highest number of refusals to the ones that were associated with the lowest number of refusals. The highest percentage of refusals was recorded when diagnostic codes J01-J04 were used (represented by the dark bars). The codes refer to acute sinusitis, acute pharyngitis, acute tonsillitis as well as acute laryngitis and tracheitis.

### Estimation of the sick leave length

The analysis of the relationships between the sick leave length as well as the patients’ age and gender was performed on a sample of 400,090 patients who excepted medical exemption. Linear regression analysis was used. The patients’ age and gender were analyzed as predictors. The acquired regression coefficients are presented in Table 4.

**Table 4:** The results of the linear regression analysis performed in the model in which gender and age were analyzed as predictors of the length of sick leave expressed in working days in the group of patients that decided to comply with the doctor's recommendation and take a sick leave

Predictors	*B*	*Beta*	*t*	*P*
(Constant)	6.94		142.65	.001
Age	.05	.06	38.61	.001
Gender	−2.64	−.15	−97.66	.001

*B* – unstandardized regression coefficients; *Beta* –standardized regression coefficients; *t* – statistical test for predictor's significance; *p* – statistical significance.

The analyzed model was statistically significant, *F*(2;399,637) = 5326.41, *p* < .001.

The relationships between the patients’ age, gender and the length of sick leave were statistically significant. The periods of sick leave were longer in the group of women. The length of sick leave correlated positively with the patients’ age. The older the patients, the longer the periods of sick leave.

The acquired regression coefficients were used to estimate the possible length of sick leave in the group of patients that refused to take it. The equation based on the unstandardized regression coefficients was:

Length of sick leave in days = .05 * Patient's age – 2.64 * Gender + 6.94.

In the equation, gender is to be coded as 0 in the case of women and 1 in the case of men.

The gender and range of participants’ age in the group of patients that refused to take a sick leave were substituted for the equation acquired in the group of patients that decided to agree to take the leave.

The estimated number of days spent on sick leave in the group of patients that refused to take it, assuming they made a different decision and complied to it, was in the range between 5 and 10 days (*M* = 7.72; *SD* = 1.35).

## DISCUSSION

In our study, the percentage of professionally active people who decide to not take a break when they are not healthy and refuse to take a sick leave after being examined by a doctor is relatively high, 27.4%, which means that more than one quarter of all professionally active people decide not to comply. One should notice that this estimation is based on a very large sample. Basing the estimation on 550,360 patients, assuming that the size of the adult population is 31,500,000, makes the statistical error negligible. However, there is a great need for further research. In other countries, the prevalence of presenteeism seems to be even higher. In the United Kingdom, 90% of British employees say that they have come to work when feeling ill [17]. The prevalence of presenteeism in Montenegro, Slovenia, Malta, Denmark and Sweden is above 50%. In Italy, Portugal and Bulgaria, it is estimated between 23–25% [18].

Research projects that investigated the relationship between presenteeism and gender yielded contradictory results. Some proved that more women were involved in presenteeism than men [11], while other that presenteeism is more frequent in the group of men than in the group of women [19]. In our study, the number of patients who decided to refuse to take a sick leave was also slightly higher in men than in women; however, the coefficient was weak, and the periods of sick leave were longer in the group of women as well as older patients.

The concept of presenteeism seems to be complex and different approaches to this complicated issue can lead to different conclusions. In the current study, a good practice could be making a comparison between the broader diagnostic categories included in the chapters of ICD-10 and specific diagnostic categories, i.e. diagnostic codes. Looking at the prevalence of presenteeism in the broader categories, one can see that patients refused to take a sick leave mainly after minor accidents. This also means that people who chose to be professionally active despite their symptoms, mainly pain, do not spread contagious diseases. Attending work while experiencing minor discomfort, even with reduced productivity, may be beneficial compared to being absent.

The analysis of the specific codes leads to a different conclusion. The most frequent categories in which presenteeism takes place are acute sinusitis, acute pharyngitis, acute tonsillitis as well as acute laryngitis and tracheitis. They all are consequences of bacterial or virus infections, which are possibly contagious. In this case, presenteeism leads not only to a loss of productivity of the people that cope with the disease, but also to a loss of productivity and possibly absenteeism of other people that would get infected. Pro-health attitudes of senior and line managers, including the promotion of work health in the workplace culture, could limit this issue; however, more research is needed to verify their effectiveness.

Using more general diagnostic categories means that more detailed categories are combined, and one looks at the same phenomenon from a different perspective.

The relationship between presenteeism and wages remains unclear. Johns found that higher wages are negatively correlated with absenteeism [20]. The results of Aronsson *et al*. [12] indicated that presenteeism is negatively correlated with the size of salary; however, Hansen & Andersen did not confirm this conclusion [21]. Since the Polish accession to the European Union, the average wage in the national economy has almost doubled (2004–2290 Polish zloty (PLN); 3rd quarter 2017–4256 PLN), and the minimum wage nearly tripled (2004 - PLN 824; 2017 - PLN 2100), one could speculate that these phenomena might partially explain the level of presenteeism observed in our study.

The correlations of being professionally active in spite of being ill can be divided into five categories, which are personality traits, attitudes, organizational policies, job design features and culture. Conscientiousness was found to be positively related to presenteeism [22]. Higher self-efficacy translates to a higher level of presenteeism [23]. Identification with professional activity strengthens presenteeism [24]. The policy of taking a disciplinary action after repeated absences can induce presenteeism [13]. Downsizing is another possible reason in the group of temporary employees, which are most likely to lose their jobs [25]. Having no permanent employment itself is also a predictor of presenteeism [26]. It was also tested as a predictor of presenteeism in longitudinal research [27]. An attempt to explain which of these phenomena may have a dominant impact on presenteeism in Poland will be the subject of further research.

An estimation of the precise costs of presenteeism would involve using a form of self-estimation of productivity loss. There are at least three approaches: estimation of productivity loss in hours (alternatively, respondents may be asked to estimate extra hours that would be needed to compensate for inefficient hours), estimation of perceived percentage loss (the most widely used), and a comparison between the productivity loss obtained from an individual and that obtained from a healthy colleague in a similar role [28, 29]. Having obtained a measure of productivity loss and taking into account the costs and benefits of working in full health, one can convert the measure into a monetary estimate. Conducting research based on both the medical records and self-measured productivity loss, even if the standardized questionnaires so far showed very limited convergent validity, could lead to very accurate, valid and relevant estimations. One of the most important drawbacks of measuring work loss due to presenteeism is the potential for common method variance, which is a consequence of asking people to self-diagnose their health and then estimate its impact on their own productivity. A research project that would combine self-measure techniques and medical records would help to resolve the problem.

Almost all medical and organizational literature treats presenteeism negatively with regard to the organization and to the employee. However, limiting the understanding of the phenomenon only to productivity loss is unduly restrictive.

The calculations carried out in the present paper allowed for a conclusion that if the patients decided to take a sick leave, they would be absent from work for a period of 5 to 10 days. Taking the type of health problem and type of job into account, presenteeism may or may not be beneficial from the productivity point of view. The most important step would be to base the decision individually in each case while being aware of all the arguments for continuing professional activity as well as of all the arguments against presenteeism. From the organizational point of view, being aware of the complexity of the issue could lead to solutions allowing for such individual decisions to be made.

## MATERIALS AND METHODS

Most research concerning presenteeism is based on data gathered with the use of self-report questionnaires. Participants estimate the scale of their presenteeism and the amount of productivity loss using, for example, percentage rates. The current study is based on medical records, which are at the disposal of The Polish Social Insurance Institution and are stored in the electronic medical record of a network of 200 clinics located in large Polish cities.

The main indicator of presenteeism in the current study is the refusal to take a sick leave in response to doctor's recommendation. The refusals are recorded in an appropriate medical database. This indicator has the value of being more objective, because patients refuse or accept to take a sick leave after being examined by a medical doctor and not just on the subjective sense of one's health.

The current study is based on data concerning 550,360 patients aged 19–64 (*M* = 35.75; *SD* = 9.97) who were professionally active, either employed or running their own businesses in 31 different cities around the country.

The records contained data on 340,759 women (61.9% of the total sample) and 209,601 men (38.1% of the total sample). They were consulted in one of 150 outpatient clinics in Poland operated by Lux Med. Ltd in the period from 20.10.2014 to 31.12.2015. All clinics used in the study had a unified electronic medical record system with the same method of data input on sick leave proposition. Patients were reimbursed by various methods of payment: national or private health insurance, and out of pocket, but individual information was not provided. Each record included information about the age, gender, diagnosis in terms of International Statistical Classification of Diseases and Related Health Problems (ICD-10) chapter, diagnosis in terms of ICD-10 code as well as the patient's reaction to the doctor's recommendation on sick leave (coded dichotomically, either complying or refusing).

Statistical analysis of consisted of appropriate statistical tests and effect size measures. Statistical tests were used to verify statistical significance, ie. to verify if associations exist and effect size measures were used to measure their strength. Statistically significant results are those which are unlikely to be results produced by chance. Results acquired in big samples can be statistically significant, but still quite weak and practically unimportant. The effect size measures allow to ascertain if the acquired results are practically meaningful. Practical significance is inferred from the size of the effect while statistical significance is inferred from the precision of the estimate. Associations between refusals to take a sick leave and the patients’ age, gender, diagnosis in terms of ICD-10 main categories and detailed codes were tested with the use of the Pearson's chi-squared test of independence. The analysis was supplemented with the Cramer's V effect size measure.

The results of the analysis of relationships between the length of sick leave (expressed in the number of days) performed with the use of linear regression analysis on data acquired from patients who decided to accept sick leave were further used to estimate the possible length of sick leave in the group of patients that refused to take it. This would allow to consider the scope of absenteeism that would have to be taken into account if the patients made a different decision and accepted sick leave.
